# Orthopaedics and Rheumatology on the Move

**Published:** 2014-01

**Authors:** David Warwick

This is a rather unique and appealing book. It has been written by three junior doctors, who have summarised orthopaedics and rheumatology into a very concise pocket sized book. To make it even more portable, there is a scratch code allowing a single digital version to be downloaded on to the purchaser’s tablet or mobile phone.

Although the book is short (just 250 pages), it is extremely well designed and structured. Headings, subheadings, colours, fonts and bullet points are used to convey logically, clearly and concisely all the key facts without encumbrance by unnecessary prose. Crucial points and summaries are separated into blue boxes, which the authors call ‘micro facts’; important details are separated into red boxes, which they call ‘micro prints’. The book is illustrated liberally with diagrams, radiographs and clinical photographs. There are dozens of clinical cases. At the end, there is a clinical quiz to test the reader’s newly found knowledge and insight. The amount of knowledge imparted by this effective format is remarkable.

The book would be ideally suited to those who want an enjoyable and simple way of grasping the very basics of orthopaedics and rheumatology. It would also suit those who need to revise the essentials of these topics, having learnt from bigger tomes and clinical experience. Medical students, junior doctors, general practitioners, nurses and physiotherapists will all find this a worthwhile purchase. There is not enough for orthopaedic or rheumatology trainees, who need detail on surgical techniques and medical treatment. Nevertheless, even these trainees would enjoy the book as a simple revision text to reassure them of their knowledge, to clarify their understanding and to help them see the wood for the trees. They are bound to pick up many valuable facts that they had previously overlooked or realise the importance of something that they had not previously realised. I did.

